# X-ray investigation of lateral hetero-structures of inversion domains in LiNbO_3_, KTiOPO_4_ and KTiOAsO_4_

**DOI:** 10.1107/S2053273315001503

**Published:** 2015-03-13

**Authors:** Thomas S. Lyford, Stephen P. Collins, Paul F. Fewster, Pamela A. Thomas

**Affiliations:** a Panalytical Research, The Sussex Innovation Centre, Falmer, Brighton, BN1 9SB, England; b Diamond Light Source Ltd, Diamond House, Harwell Science and Innovation Campus, Didcot, Oxfordshire, OX11 0DE, England; c Department of Physics, University of Warwick, CV4 7AL, England

**Keywords:** ferroelectrics, diffraction, coherence, synchrotron radiation, grating

## Abstract

In this paper periodically domain-inverted (PDI) ferroelectric crystals are studied using high-resolution X-ray diffraction. Rocking curves and reciprocal-space maps of the principal symmetric Bragg reflections in LiNbO_3_ (LN) (Λ = 5 µm), KTiOPO_4_ (KTP) (Λ = 9 µm) and KTiOAsO_4_ (KTA) (Λ = 39 µm) are presented. For all the samples strong satellite reflections were observed as a consequence of the PDI structure. Analysis of the satellites showed that they were caused by a combination of *coherent* and *incoherent* scattering between the adjacent domains. Whilst the satellites contained phase information regarding the structure of the domain wall, this information could not be rigorously extracted without *a priori* knowledge of the twinning mechanism. Analysis of the profiles reveals strain distributions of Δ*d*/*d* = 1.6 × 10^−4^ and 2.0 × 10^−4^ perpendicular to domain walls in KTP and LN samples, respectively, and lateral correlation lengths of 63 µm (KTP), 194 µm (KTA) and 10 µm (LN). The decay of crystal truncation rods in LN and KTP was found to support the occurrence of surface corrugations.

## Abstract 2

Periodically-poled ferroelectric crystals are studied by observing their superlattice (grating) diffraction profiles with high-resolution X-ray diffraction. In order to successfully model the data, the effects of strain, and sample and beam coherence, must be taken into account.

## 1. Introduction   

Ferroelectrics are non-linear dielectrics that exhibit spontaneous alignment of electric dipoles. In LiNbO_3_ (LN), KTiOPO_4_ (KTP) and KTiOAsO_4_ (KTA), the electric dipoles are aligned with the direction of a polar axis within the structure, and application of a suitable electric field causes the dipoles to reverse their direction, thus switching the sense of the spontaneous electrical polarization in the crystal.

The crystals studied in this paper all exhibit second-harmonic generation (SHG) – the process whereby monochromatic light incident upon the crystal emits radiation of twice the incident frequency. By periodically modulating the sense of the spontaneous polarization within the crystal by means of a patterned electrode (Armstrong *et al.*, 1962 ▶; Fejer, 1994 ▶; Houé & Townsend, 1995 ▶), the incident and scattered radiation are quasi-phase matched, allowing good wavelength conversion efficiencies. These periodically domain-inverted (PDI) crystals can then be employed in a wide variety of optical devices.

Though the production of PDI crystals is relatively straightforward in principle, fundamental questions surrounding the atomic level physics of the structural inversion remain unanswered. For example, it is unknown whether the pivot atom between adjacent domains is defined by the orientation of the domain walls, or whether the thickness of the domain walls changes from the surface to bulk regions. KTP and its isomorphs pole anisotropically, favouring domain growth along [010] on the conventional 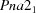 setting of the cell with *a* = 12.819 (3), *b* = 6.399 (1), *c* = 10.584 (2) Å (Thomas *et al.*, 1990 ▶). Gratings of good integrity were produced with walls designed to be parallel to (100) planes (*a*-poled), whereas the *b*-poled gratings with walls parallel to (010) were of a poor quality. This anisotropy is explained in terms of the Miller–Weinreich model (Miller & Weinreich, 1960 ▶).

X-ray topography, which can investigate the surface and bulk, has been used to study PDI LiTaO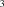 and estimate domain-wall widths of a crystalline sample in the bulk (Kim *et al.*, 2000 ▶). Results from the literature are summarized in Tables 1 ▶ and 2 ▶. Topography, whilst being the most strain-sensitive technique and allowing access to the bulk, is limited by the resolution of the detector (the minimum grain size of the plate/film or pixel size), which at present is ∼1 µm. Alternative techniques suggest the domain-wall widths in LN and KTP are at, or below, the topographic resolution limit. It therefore appears that another technique must be used to measure the domain-wall thickness in the bulk. High-resolution transmission electron microscopy (TEM) can provide a resolution of nm, but is not ideal as it limits the thickness of the PDI to 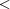 10 µm, and may cause an accumulation of charge at the domain wall, distorting its shape by straining it (Wick & Lewis, 1968 ▶). High-resolution X-ray diffraction (HRXD) has been used here as an alternative to investigate the scattering properties of the PDIs in question.

### 1.1. Samples   

This work employed three samples, LN, KTP and KTA, with different lateral periods. All of the sample plates, which were *z*-cut before poling, were poled using the bulk electric field method (Myers *et al.*, 1995 ▶; Rosenman *et al.*, 1999 ▶) until domains traversed the entire plate thickness, then polished to a high optical quality to remove surface scratches.

The LN sample was poled at Southampton University (Ross, 2000 ▶). The plate, which had dimensions of 10 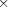 30 mm and a thickness of 0.55 mm, was poled with domain walls normal to [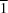
20], over an area ∼ 7 × 7 mm with a grating period of Λ = 5.01 µm. The crystal was then etched, revealing no domain broadening and duty cycle 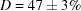.

The KTP sample was grown and poled at Tel Aviv University (Rosenman, 2002 ▶). The plate dimensions were 8 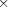 4 mm with a thickness of 0.4 mm. The plate was low-temperature *a*-poled over an area ∼ 3 × 3 mm with a grating period of Λ = 9.02 µm. Etching revealed no domain broadening and a duty cycle of 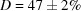.

The KTA sample was grown and poled at Tel Aviv University (Rosenman, 2002 ▶). The plate dimensions were 4 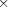 4 mm with a thickness of 0.53 mm. The plate was low-temperature *a*-poled over its entire area with a grating period of Λ = 38.85 µm. Etching revealed no domain broadening and a duty cycle of 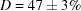.

## 2. Data collection   

The data presented were all collected at SRS Station 16.3, Daresbury Laboratory (Collins *et al.*, 1998 ▶), at an energy of 15 keV. Wavelength selection was made by a vertical four-bounce (+,−,−,+) Si 111 channel-cut crystal monochromator reflecting from the 333 planes. Calibration of the beam energy was provided by scanning a platinum foil over the Pt 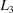 edge, 11.564 keV. The monochromator is 34 m from the source – a 6 T wiggler – which has FWHM electron-beam dimensions of 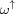 = 0.4 mm and 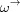 = 1.5 mm; thus diffraction was in the vertical plane to exploit the better beam coherence. The Eulerian cradle upon which the samples were mounted could be rotated in ω, ϕ and χ (Collins *et al.*, 1998 ▶) with a sphere of confusion of radius 25 µm. Angular selection of the diffracted beam was provided by an Si 111 channel-cut analyser in single- or three-bounce (−,+,−) modes. The incoming beam flux was recorded by a custom-built xenon-filled ion chamber and the diffracted flux by a modified Bede Extended Dynamic Range scintillator. The data-acquisition system used is a combination of PINCER, a general-purpose command interpreter, and the macro library CLAM (Collins *et al.*, 1998 ▶), which provides a uniform interface to all hardware controls *via* a set of virtual motors.

**Figure 1:**
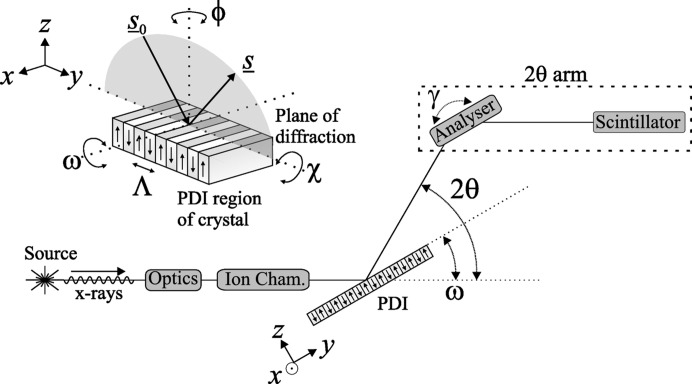
Schematic of the sample orientation and diffraction geometry.

Samples were mounted accurately with a minimum amount of strain by using a flat glass block and a very small amount of wax on one of the *z* faces. The glass block was attached to the goniometer head which was mounted on the diffractometer cradle. The samples were oriented such that the plane of diffraction fell normal to the domain walls, as shown in Fig. 1 ▶. The X-ray beam passes through the centre of rotation of the cradle and the station has a video camera with cross-hairs trained on this position. Thus, once the sample was mounted it was possible to know the position of the beam on the sample, and the dimensions of the sample and PDI region with reference to the virtual motors which translated the sample.

Etching of the samples demonstrated a sometimes uneven quality of domains across the sample surface. Thus, using the cross-hairs, the beam footprint was aligned on regions displaying good uniformity. Further, to ensure data were collected from a region with good surface and PDI qualities, the beam was rastered over the surface of the PDI. In this way diffraction profiles of a particular reflection were collected in a grid over the PDI region until an area which displayed well developed satellites was identified. A smaller grid was then employed to optimize this position. At this point all the axes ω, ϕ, χ and γ in (−,+,−) mode were optimized before the diffraction profile was recorded. Typically the ω increment in the scan was the minimum step size possible, 0.1 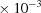
°, and counting times were adjusted according to the scattered flux. The large size of the LN sample relative to its PDI region meant that it was also possible to collect rocking curves on the untreated region of this sample.

Reciprocal-space maps were collected using a single bounce from the analyser crystal, using two different procedures. The first collected successive 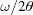 scans (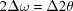) about different sample positions. The second method reduced the time taken recording a map by rocking the analyser crystal rather than moving the detector arm, as very small analyser rotations, 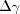, approximate to detector movements, 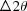. The ranges over which ω and 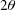 were varied were chosen so that the reciprocal-lattice point (r.l.p.) would sit centrally in the map.

### 2.1. Reflections of interest   

Imaging work on similar ferroelectric crystals (Hu *et al.*, 1996 ▶, 1998 ▶; Rejmánková *et al.*, 1998 ▶; Pernot-Rejmánková *et al.*, 2000 ▶) suggested that the most likely means of seeing effects from adjacent domains would be to diffract from Friedel pairs which exhibited large phase differences, 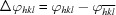. Experimentally, however, symmetric Bragg reflections were preferred as asymmetric reflections can exhibit complex absorption effects. Thus, planes parallel to the surface of the samples, 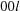 reflections, were studied.

The magnitude of the phase difference between adjacent domains is dependent on the pivot atom linking the inverted structures. In *a*- and *b*-poled LN, this pivot atom is unknown. The atomic positions in ferroelectric LN are: Li (0, 0, 0.2787); Nb (0, 0, 0); O (0.0476, 0.3433, 0.0634). The structure possesses a pseudo-twofold axis along the [100] direction which passes through the O atom at (0.3809, 0.0100, 0.7301). Using this pseudosymmetry it has been proposed that the twinning between adjacent domains could take place through the O atoms, though this has not been verified (Hu *et al.*, 1996 ▶). It is also possible to twin through the Nb atom. Both possibilities were investigated here to see whether the pivot atom could be distinguished using HRXD. For [100] domain walls, KTA is known to twin through the As1 atom (Pernot-Rejmánková *et al.*, 2000 ▶) and KTP through the P1 atom (Rejmánková *et al.*, 2003 ▶). Table 3 ▶ shows the reflections which were used, the associated phase differences and average scattering factors of the Friedel pairs involved.

## 3. Results and analysis   

The most noticeable feature in most of the diffraction profiles is the occurrence of satellite (or superlattice) peaks, with angular spacings given by 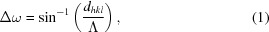
which is equivalent to 1/Λ in 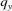. Thus, the satellites are identified as being caused by features with a period of Λ, and so are attributed to the PDI grating on each sample. The profiles were fitted using a modification of the basic pseudo-Voigt curve, where the Gaussian and Lorentzian widths are uncorrelated. The algorithm was constrained so that every peak had the same (Gaussian and Lorentzian) widths and then iterated 400 times.

All of the FWHMs from the ω scans and reciprocal-space maps of the satellites or central peaks displayed in the figures can be found in Table 4 ▶, as can the theoretical Darwin widths. Data from the untreated region of the LN sample (not shown) are also included in Table 4 ▶. Sample strain is derived by the modification of the peak width in the 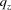 direction, from its theoretical value after correcting for instrumental broadening, 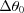 (the Darwin width), according to Cauchy’s relation (Warren, 1969 ▶): 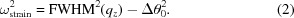


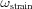
 was substituted into the derivative of Bragg’s law to give the distribution of strains present within the sample, 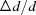. 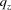 scans were not collected for all of the reflections under investigation, so it is also necessary to contrast the FWHMs of the recorded ω scans with 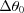. Peak widths in 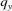 are far less simple to analyse as both contain reflection-order-dependent sample and instrumental broadening effects. As calculation of these contributions is non-trivial, it is more useful quantitatively to compare these with 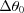, or with ω-scan FWHMs collected on and off the LN PDI region to assess the effect on the crystal.

Dead-time losses in the data were accounted for by linearly varying the beam flux whilst recording the ion-chamber and scintillator count rates. These data were most accurately fitted using the *non-paralyzable* model (Bowen & Tanner, 1998 ▶). The dead-time-corrected scintillator count rate of each scan was divided by the ion-chamber count rate to normalize the intensity.

**Figure 2:**
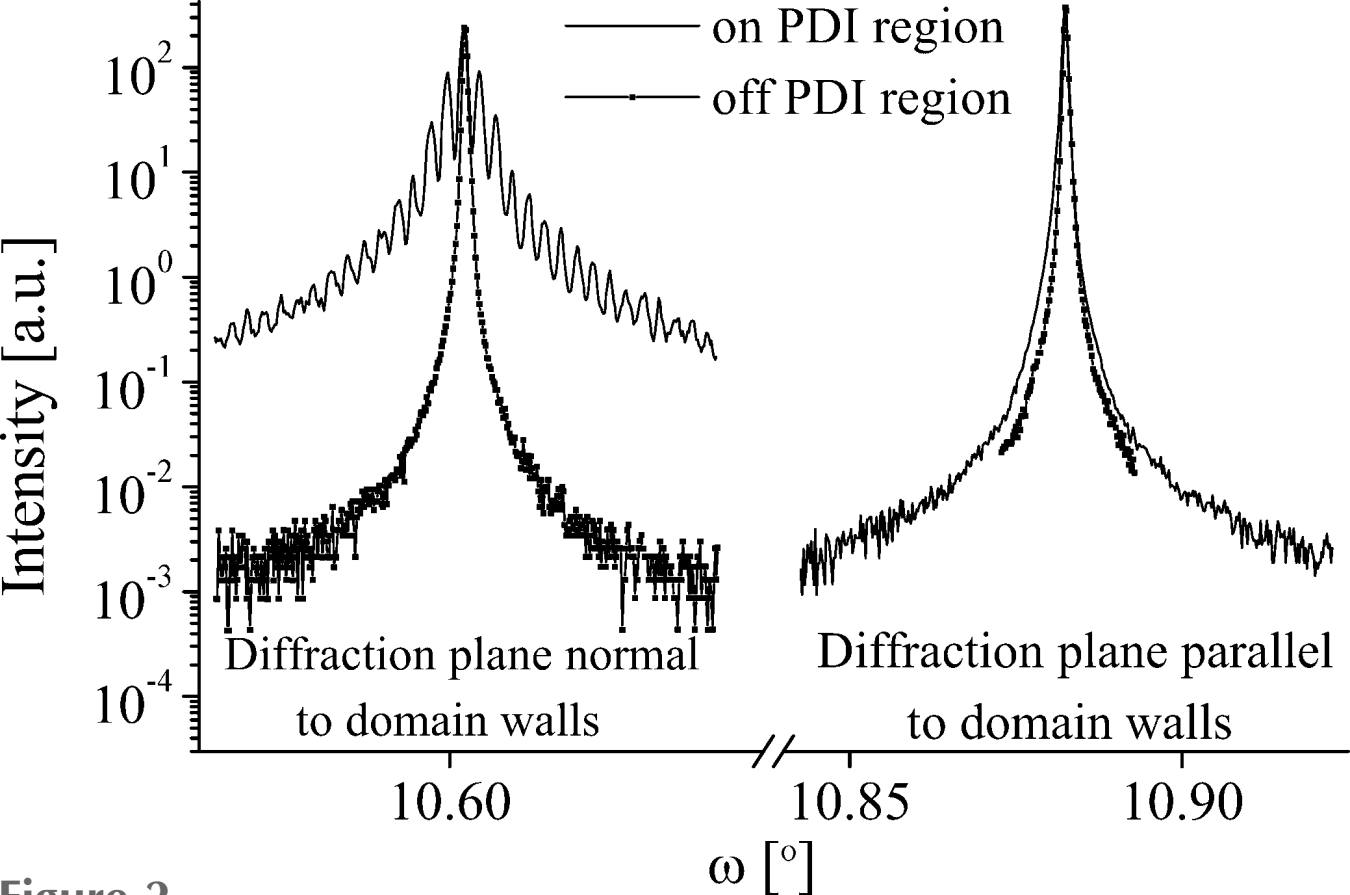
Triple-axis triple-bounce diffraction profiles at 15 keV of the 006 reflection on different parts of the 5.01 µm LN sample.

Fig. 2 ▶ shows the effect of the grating region on the LN 006 diffraction profile by displaying scans taken on and off the grating with the domain walls parallel and perpendicular to the diffraction plane. The profile of the latter on the PDI shows 14 pairs of well resolved satellites, symmetrical and reducing monotonically about 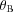. The fact that the FWHM of the grating peak parallel to the domain walls is very similar to the peaks (at ϕ = 0° and 90°) on the untreated region implies that the sample is relatively unchanged parallel to the domain walls. The large increase in the FWHM and integrated intensity when scattering perpendicular to the domain walls suggests that the poling process appears to have significantly damaged the structural quality of the grating region in this direction.

**Figure 3:**
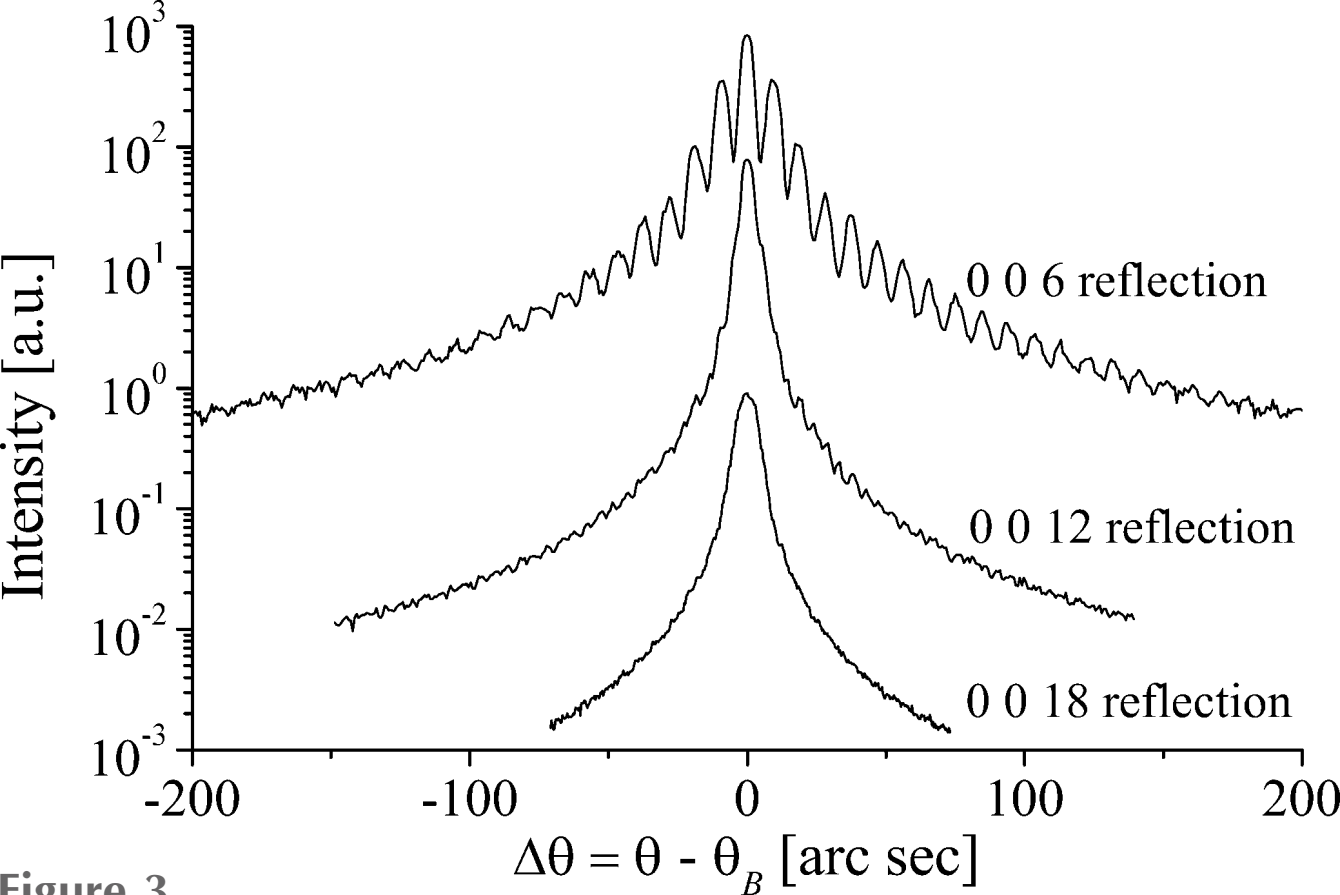
Diffraction profiles of symmetric Bragg reflections on the PDI region of the LN sample (profiles vertically offset for presentation).

Fig. 3 ▶ shows the (006*n*) reflection series. The 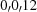 reflection shows only very weak satellites and 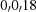 none at all. Both the central peak and satellites are significantly broader than on the untreated region of the sample. The increase in the FWHM and the integrated intensities on the grating region compared to the untreated region suggests that the poling process has caused structural damage.

**Figure 4:**
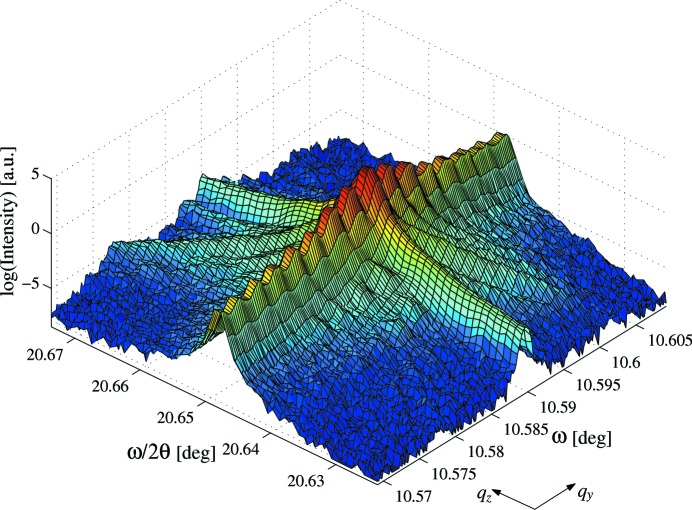
Triple-axis single-bounce reciprocal-space map of 006 reflection at 15 keV of the 5.01 µm LN sample.

The LN 006 reciprocal-space map shown in Fig. 4 ▶ has a small angular range and so axes of ω *versus* 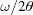 approximate to 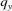 *versus* 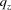. The grating structure manifests itself as satellites in the 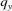 direction. The width of the main peaks in 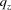 is FWHM(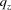) = 8.13 arcsec, which corresponds to a strain distribution of 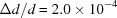 on the grating. The behaviour of the crystal truncation rod (CTR) was fitted using (Andrews & Cowley, 1985 ▶) 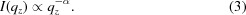
Fitting revealed 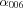 = 2.43 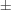 0.07, indicating that the grating region has a less than perfect surface, consistent with the surface corrugations observed in periodically poled LN (Rommeveaux, 2002 ▶). A diagonal analyser streak can also be observed.

**Figure 5:**
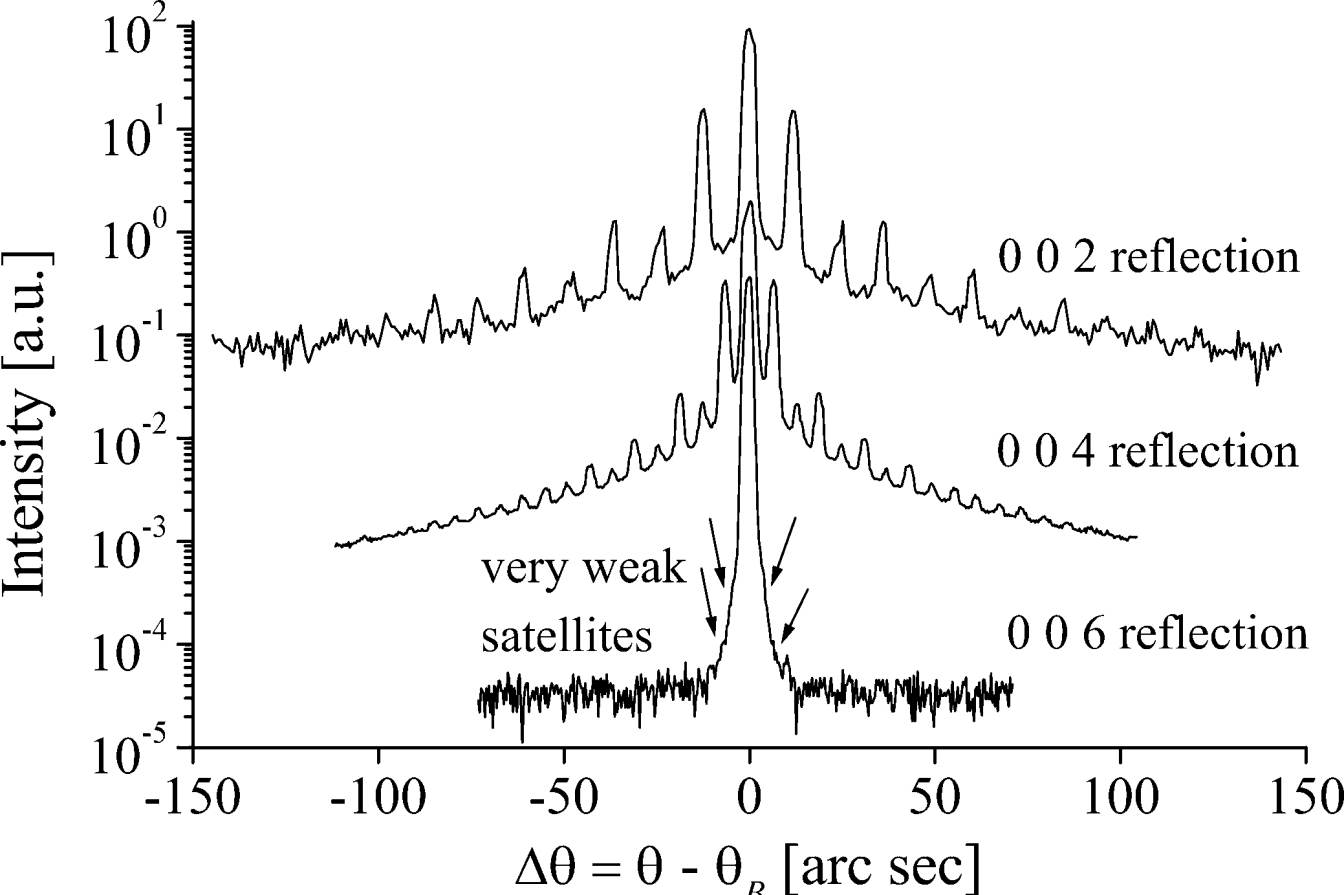
Diffraction profiles of symmetric Bragg reflections in the PDI region of the KTP sample.

Fig. 5 ▶ shows the effect the increase in the (002*n*) reflection series has on the KTP diffraction profiles. Strong, well resolved satellites can be seen in the 002 and 004 reflections. Despite a wide search of the grating no strong satellites could be found for the 006, though very weak satellites are indicated on the figure, which have angular spacings given by equation (1), showing they are due to the grating. For the 002 and 004 reflections, the first three even satellite pairs are suppressed in intensity relative to the odd, which was not observed in the LN sample.

**Figure 6:**
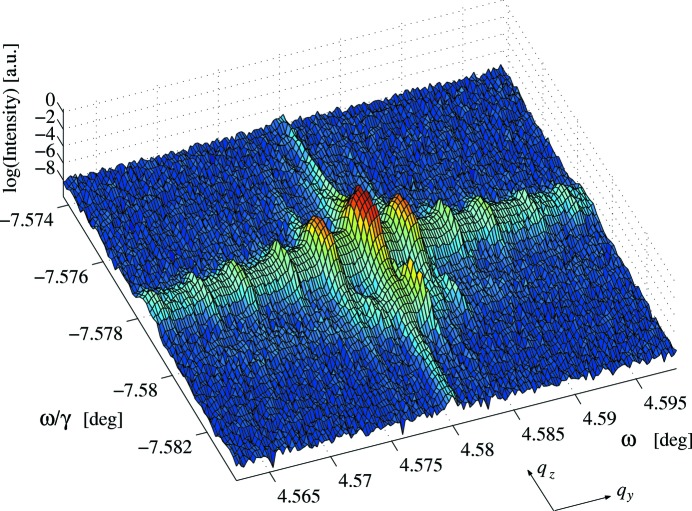
Reciprocal-space map of 002 reflection from the KTP sample.

The KTP 002 reciprocal-space map shown in Fig. 6 ▶ was collected by rocking the analyser through 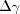. The width of the main peak in the 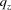 direction is FWHM(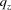) = 2.58 arcsec, which is very much broader than the Darwin width, 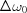 = 0.17 arcsec, giving a strain distribution of 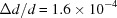 on the grating region. The CTR has been fitted with equation (3), giving 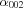 = 1.98 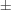 0.17, suggesting the surface is relatively perfect. No analyser streak is seen. A second weaker set of peaks offset from the first in both 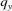 and 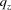 is shown, most likely from a different part of the PDI pattern.

**Figure 7:**
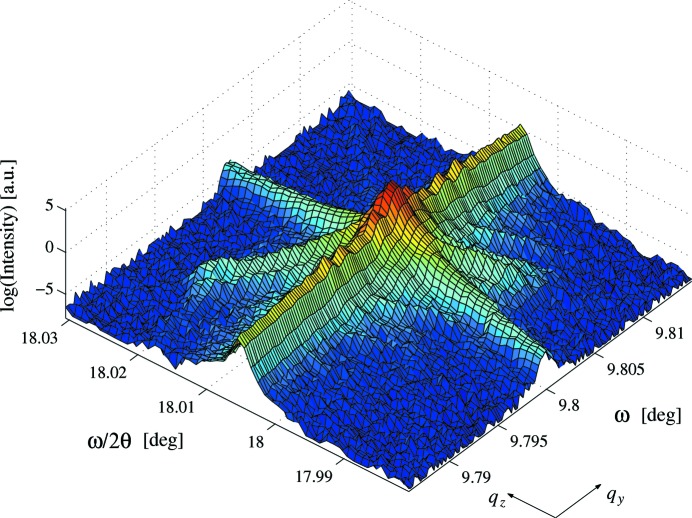
Reciprocal-space map of 004 reflection from the KTP sample.

The width of the 004 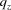 (Fig. 7 ▶) is FWHM(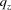) = 5.94 arcsec, significantly broader than 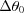 = 1.90 arcsec, giving a strain distribution of 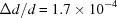. This is in good agreement with the strain distribution found in the 002 map. Fitting of the CTR gives 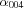 = 2.58 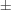 0.20 however, which is not in good agreement with 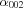 = 1.98. It is possible that the 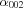 result is anomalous as the subsidiary peaks shown in the 002 map would contribute intensity from different regions in *q*-space to the point of measurement. Thus, the decay of the CTR in the 002 map may not be as fast as would be expected in the 004 map. Again a strong analyser streak can be seen.

**Figure 8:**
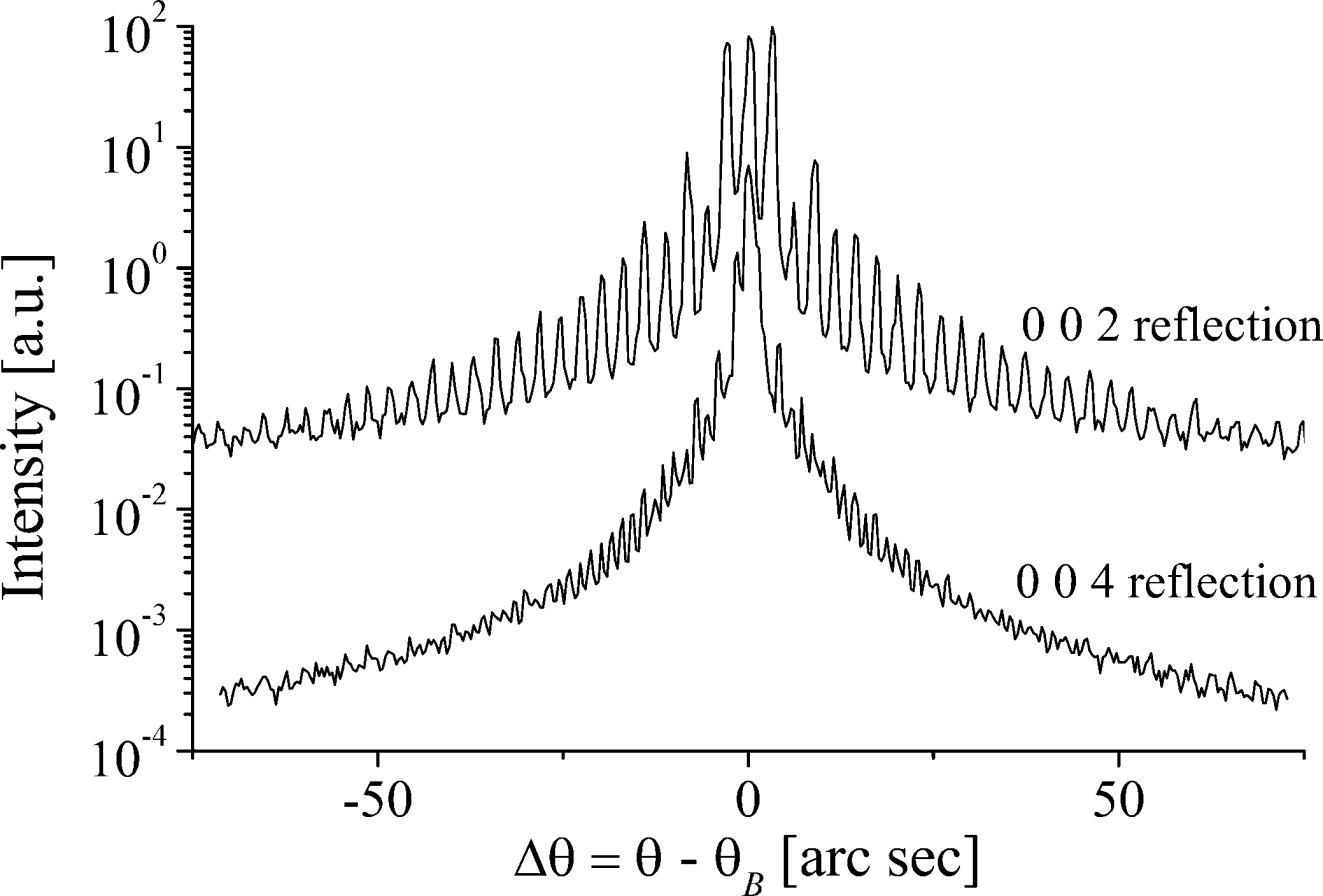
Diffraction profiles of symmetric Bragg reflections in the PDI region of the KTA sample.

Fig. 8 ▶ shows the effect the increase in the (002*n*) reflection series has on the KTA diffraction profiles. Both reflections show over 20 pairs of very strong, though poorly resolved satellites. This is most noticeable for the central peak of the 002 reflection, which is smaller than the first high-angle (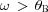) satellite. Though the resolution is barely adequate to resolve the satellites, it is still sufficient to show that the first two pairs of even satellites are suppressed in intensity relative to the odd. This was something also noted for the 9.02 µm KTP 002 reflection. For the 002 and 004 reflections, the first three even satellite pairs are suppressed in intensity relative to the odd, something not observed for the LN sample.

Without a surface mapping it is impossible to estimate either the surface quality of the grating, or the strain distribution present in the grating. It is noteworthy that the FWHM of the satellites, 0.9 arcsec, is smaller than that of the Darwin width of 1.9 arcsec.

## 4. Summary of the diffraction profile results   

### PDI LiNbO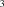   

4.1.

Previous Bragg–Fresnel (BF) imaging suggested that it is the Friedel-pair phase shifts, 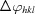 (Table 3 ▶), between the adjacent domains which give rise to the satellites. The results showed that only the 006 reflection, 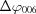 = 63.5° for Nb as pivot atom or 106.4° for O as pivot, displayed well resolved satellites, with 15 satellite pairs being visible. Very weak satellites could be seen in the 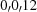 reflection (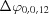 = −34.6° or −53.7°), and the FWHM of the 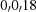 reflection (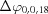 = 20.6° or −30.0°) implied that satellites were present, though it was not possible to resolve them. If indeed it is the phase shift between the adjacent domains which causes the satellites, these results suggest that only large phase shifts can be used to study grating structures using this technique.

The decay of the dynamical streak in the 006 reflection reciprocal-space map is 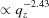, showing the sample surface on the grating is non-perfect, in agreement with the corrugations observed in periodically poled LN (Table 2 ▶). This is in agreement with the comparison of ω-scan widths and integrated intensities taken on and off the grating, which all showed the grating peaks to be significantly broader and more intense, suggesting kinematical scatter from a damaged structure. The width of the 006 reflection in 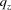 gave a strain distribution of 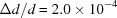 on the grating, in direct agreement with Yang & Mohideen, who estimated a shear strain perpendicular to a domain wall in the LN analogue, LiTaO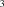, of 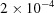 (Yang & Mohideen, 1998 ▶). The strain distribution on the grating appears confined to the direction normal to the domain walls, as 006 reflection profiles collected whilst scattering parallel to the domain walls displayed FWHMs unchanged from the untreated region, and only slightly higher diffracted intensities.

### 4.2. PDI KTP   

Reference to the profiles and the associated Friedel-pair phases (Table 3 ▶) supports the idea that satellites may be linked with the phase shift, 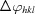. The 002 and 004 reflections have large phase shifts of 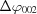 = 106.0° and 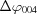 = −53.7°, and both display very strong, well resolved satellites. The 002 and 004 reflections displayed ten and 14 satellite pairs each, respectively, although only the first five satellite pairs were strong and well resolved for the 002 reflection. The 006 reflection has a small phase shift, 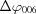 = 5.7°, and only showed three to four very weak satellite pairs which could not be resolved. Thus, the larger the phase shift, the larger the magnitudes of the satellites.

There appears to be a large difference between the satellite behaviour in the 5.01 µm LN profiles in comparison to the 9.02 µm KTP profiles. Strong, well resolved satellites are found for the KTP 004 reflection, but not for the LN 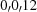 reflection, though they both have a similar phase shift, 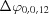 = −34.6° (for Nb pivot atom) or −51.7° (for O pivot atom), whereas 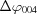 = −53.7° for the KTP 004 reflection. The suppression of the first three even satellite pairs relative to the odd is also something noted in the KTP 002 and 004 reflections, but not in the LN 006 reflection. As the phase shifts of the 002 and 004 KTP reflections are similar to both possibilities of the LN 006 phase shift, but not to one another, it is inferred that it is a property of the sample itself, and the phase shift, which influences the relative satellite intensities. Thus, there may be a different mechanism for satellite generation in 9.02 µm KTP in comparison to the 5.01 µm LN.

The strain distribution perpendicular to domain walls on the grating, estimated from the 002 and 004 reciprocal-space maps, is in good agreement giving 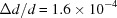 and 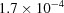, respectively. This is of the same magnitude as the strain distribution found normal to the domain walls in LN. The maps do not concur regarding the surface quality, giving 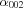 = 1.98 and 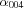 = 2.58. Surface corrugations have been observed in periodically poled KTP (Table 1 ▶), making the 004 result more likely, and it is also possible that the 002 result is anomalous due to an instrumental artefact introduced by the analyser.

### 4.3. PDI KTA   

The fact that the 002 and 004 reflections displayed satellites with angular spacings given by equation (1) proves they are due to the grating. Reference to the 38.85 µm KTP diffraction profiles and the associated Friedel-pair phases (Table 3 ▶) is in support of the link between satellite generation and phase shift, 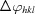. The 002 and 004 reflections have large phase shifts of 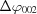 = 131.6° and 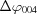 = −43.1°, respectively, and both profiles display over 20 pairs of satellites, with the 002 satellites being stronger and more resolved than the 004 satellites.

Qualitatively these scans are in agreement with the 9.02 µm KTP 002 and 004 reflections, which both displayed the suppression of the first three even satellite pairs in intensity relative to the odd. As KTP and KTA are isomorphs, this agrees with the idea that it is a property of the sample itself, as well as the phase shift, that defines the relative satellite intensities.

## 5. Discussion of the effect of beam coherence   

As the PDI material contains periodic features, the number of these features coherently diffracting will be important to the final profile. Thus, the coherence of the beam needs to be calculated. In Bragg geometry the relevant coherence length is a combination of the spatial (lateral) and temporal (longitudinal) coherence functions, dependent upon the angle of incidence, 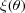. Explicit calculation of ξ at any particular angle of incidence is non-trivial as it includes many variables; however it can be estimated from 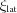 and 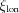. The vertical lateral beam coherence length of 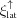 = 3.5 µm at 15 keV, with a double-bounce monochromator, is determined by the electron-beam size. However, the low divergence afforded by the four-bounce dispersive Si 333 monochromator system leads to a much larger value of 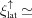 22 µm.

The temporal coherence of the beam 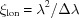, where the bandwidth of the monochromator crystal, 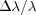, can be related to the specific Darwin width, 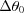, through the derivative of Bragg’s law to give 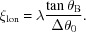
At 15 keV the Si 111 and 333 reflections provide temporal coherences of 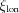 = 0.6 and 9.4 µm, respectively. These values suggest that for Station 16.3 ξ ∼ 3–22 µm, depending on the monochromator setup.

The angular dependence of ξ is analogous to the beam footprint on the sample. The coherence in the plane of the crystal surface, 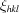, can be assumed to follow the relation
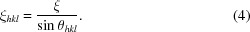
High-order reflections, where the beam is incident at steep angles, suffer from a lower fringe visibility relative to low-order, low-angle reflections where the coherence length can interact along a greater length of the sample. All of the triple-axis triple-bounce profiles recorded at 15 keV used the Si 333 monochromator, and so the coherence length is close to the upper limit of ξ = 21.8 µm. Table 5 ▶ has been generated to show the effect of equation (4) on the coherence of the beam in the plane of the crystal surface. The values suggest that once the beam coherence falls below ∼100 µm, satellites cannot be resolved. It also shows that the coherences of the KTP and KTA reflections are approximately double that of the LN reflections.

## 6. Reciprocal-space resolution   

**Figure 9:**
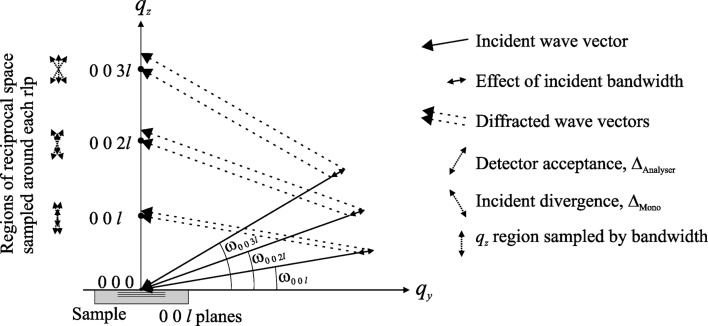
The variations of the instrumental functions in reciprocal space for different symmetrical Bragg reflections at a single wavelength.

Fig. 9 ▶ shows the incident and diffracted wavevectors for three symmetric Bragg reflections at a fixed wavelength. While the incident divergence, detector acceptance and incident bandwidth are the same in all cases, their effect on the regions of reciprocal space around the r.l.p.’s that are sampled is dependent on the reflection, as shown in the figure. The incident divergence and detector acceptance sample regions of both 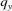 and 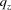, whilst the incident bandwidth only samples regions in 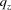. (The detector actually receives a convolution of all these functions, and as Fig. 9 ▶ is only a two-dimensional representation of a three-dimensional effect, the region of reciprocal space probed should be thought of as a volume whose size is dependent on the reflection under investigation.) Thus, the low-order reflections experience less convolution than the higher-order reflections, and so might be expected to have a better relative resolution.

## 7. Simulation   

Three methods were employed to describe the PDI as a scattering object and simulate the recorded rocking curves. The diffraction profile results contained strong features with which to compare the simulations: the even satellite suppression in 002 and 004 reflections from the KTP and KTA samples; the very weak satellites in the 006 reflection from the KTP sample; the uniform satellite intensities of the 006 reflection; and the very weak satellites of the 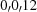 and 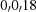 reflections from the LN sample.

Satellites from all of the samples are found at positions of 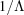 in 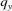, meaning that each sample contains a strong feature with a period of Λ. This feature is the structure-factor difference between the 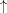 and 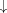 domains. This is not, however, the only periodic feature that the PDI samples contain. PDI LN and KTP have been reported to have surface corrugation depths of 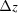 = 0.5–10 nm (Yang & Mohideen, 1998 ▶; Wittborn *et al.*, 2002 ▶; Rommeveaux, 2002 ▶) and 2–30 nm (Wittborn *et al.*, 2002 ▶; Rejmánková *et al.*, 2003 ▶), respectively. These corrugations also provide satellites with spacings of 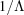. Surface domain-wall thicknesses for LN and KTP of *w* = 0.15–15 µm (Yang & Mohideen, 1998 ▶; Kim *et al.*, 2000 ▶; Wittborn *et al.*, 2002 ▶) and 20–80 nm (Wittborn *et al.*, 2002 ▶), respectively, have also been reported. The Malis–Gleiter model of a ferroelectric domain wall (Malis & Gleiter, 1976 ▶) supposes that the electric dipole rotates from 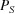 to −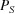 over the domain-wall width, *w*, and so the structure factor in the region of this rotation, 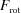, is assumed to be less than 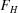 or 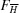, in the surrounding domains. It is also probable that 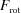 for the 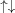 wall would be opposite for the 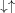 wall, and so the scattering properties of the domain walls would also provide satellites with a spacing of 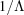.

The strain distributions derived from the LN and KTP 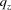 peak widths with the diffraction vector normal to the domain walls will affect the structure factor. Walls also attract space-charges and defects which contribute to the strain. Thus, it is highly probable that each domain wall contains a strain component, and as X-rays are highly sensitive to strain, the crystal will also contain a scattering feature, 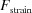, which is periodic with 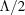 (and so giving satellites at positions of 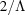 in 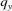).

**Figure 10:**
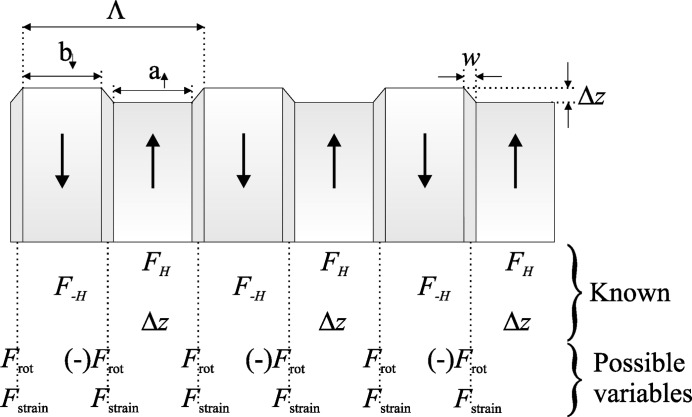
Periodic modulations within a PDI crystal to which X-rays are sensitive.

Fig. 10 ▶ summarizes the modulations existing within a PDI crystal to which X-rays are sensitive. Previous BF imaging studies showed that no domain broadening was observed in KTP, and the dielectric relaxation time of LN suggests that domain broadening is unrealistic in LN PDIs. Thus domains are considered as having a fixed width through the sample. The lateral dimensions of the 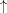 and 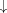 domains are denoted *a* 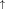 and *b* 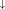, respectively, and so the duty cycle *D*(%) = 100(*a* 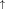/Λ).

### 7.1. Surface corrugation model   

Rocking-curve simulation has been used to characterize the geometry of the shallow corrugations on a GaAs (100) surface (De Caro *et al.*, 1994 ▶). The model used for the reflecting power, 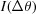, is applicable to very shallow corrugation depths where the troughs of the corrugations are always illuminated by the beam: 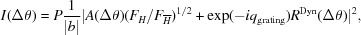
where *P* is the polarization state of the beam, *b* the ratio of direction cosines of the incident to scattered waves, 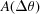 is the amplitude of the diffracted wave from the grating, 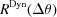 the dynamical reflectivity profile of the perfect bulk crystal below the grating (Fingerland, 1971 ▶) and 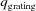 the phase modulation of the grating. This model was then used to simulate the interference fringes produced by surface corrugations in KTP, KTA and LN, using the domain-wall thicknesses and surface corrugations found in the literature. To see the effect of the surface corrugation on its own, the crystals were considered as being single domain. As 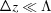, and only symmetric Bragg reflections were studied, the troughs of the corrugations would have always been illuminated, and so the amplitudes were summed. While the model produced satellites, the first two or three satellite pairs were swamped by the dynamical scattering of the bulk, and in comparison to the data, the simulated satellites were two to five orders of magnitude smaller than the recorded ones. The model was then amended so that the intensities rather than the amplitudes were summed; however this produced a poorer result. The duty cycle of each sample was also allowed to vary within its limits, though this produced no noticeable improvement. The effect of the surface corrugation on the diffraction profiles, in both the kinematical and dynamical regimes, for all the materials, was therefore shown to be negligible.

### 7.2. Semi-kinematical model   

Table 5 ▶ shows that, for every reflection investigated, the beam coherence significantly exceeds the period of the PDI grating, and the grating model of De Caro *et al.* suggested that the surface corrugations of the samples can be ignored. Thus, the beam can be approximated as being infinite, and the sample as being flat. For the case of a PDI crystal where the diffraction plane is normal to the domain walls, the complex reflection coefficient, *R*, of the reflection *H* can be defined as (Aristov *et al.*, 1990 ▶; Petrashen’ & Chukhovskii, 1989 ▶) 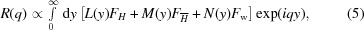
where 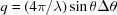 and the summation is performed over the length of the (perfectly flat) crystal surface. 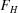, 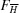 and 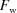 represent the structure factors of the 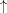 domain, 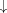 domain and wall regions, respectively. *L*(*y*), *M*(*y*) and *N*(*y*) are coefficients representing the behaviour of the structure factor in the 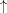 domain, 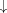 domain and wall regions, respectively. These coefficients can all be represented using a Fourier series as infinitely repeating pulse signals from 0 to 1. 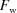 is likely to be a combination of the structure factor from the rotation of the electric dipoles, and a strain component: 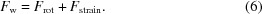
The magnitude of 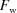 is unknown though it is likely to be of the order of 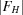. It is also unclear whether 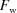 is the same for the 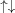 wall as the 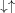 wall or not. Both possibilities were investigated using this model. Equation (5) was solved for *x* and the scattered intensity of the PDI, 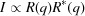, was calculated for the reflections of interest.

In all cases, simulated rocking curves of the reflections of interest contained satellites of a similar magnitude as the recorded ones, but always displayed a strong asymmetry between satellite pairs not shown in any of the data. The intensities of all the satellites were found to be heavily dependent on the duty cycle of the grating. Typically, a linear variation in Λ would cause no fixed pattern of behaviour in the satellite intensities. A linear change in the width of the domain walls, *w*, had a similar effect on the *n* = 3+ satellites, producing no clear pattern of behaviour. An increase in the scattering strength of the domain wall, 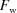, always produced an increase in the intensity of all the satellites. Several modifications of the model were made in an attempt to remove the asymmetry between satellite pairs: removing the domain wall; making the scattering strength of the 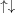 domain wall different from the scattering strength of the 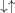 domain wall; adding intensities rather than amplitudes. All proved unsuccessful, making it impossible to produce even a rudimentary simulation which compared favourably with any of the recorded rocking curves. Thus, it was concluded that the semi-kinematical model of the PDI was not realistic.

### 7.3. Optical grating model   

This model considers the PDI as a number of intermeshed optical diffraction gratings in the Fraunhofer regime. Table 5 ▶ shows the effective coherence length of the reflections under investigation in the plane of the crystal surface, 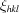, to be tens of µm in all cases. In this case, *N* = 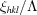 of the 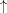 domains will be diffracting coherently with a phase of 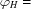 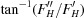, alongside *N* of the 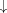 domains with a phase of 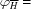 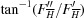, and 2*N* of the domain walls with an unknown phase. Thus, *N* can be likened to the lateral correlation length in the sample. As the gratings are coherently diffracting with themselves, the scattered amplitude from each can be considered separately, and then appropriately summed at the observation point to reconstruct the scattered amplitude from the PDI. Describing the PDI as a diffraction grating is straightforward: the number of parallel slits in the grating (an integer) is represented by the number of coherently diffracting grating repeats, *N* for the domains and 2*N* for the walls; the slit width is given by *a* 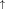 or *b* 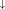 for the 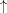 or 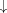 domains, respectively, and *w* for the domain walls; the slit-centre-to-slit-centre distance is Λ for the domains and 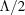 for the domain walls. The reflected amplitude from the three gratings, offset from one another at the same (distant) observation point, can be expressed (Hecht & Zajac, 1977 ▶): 
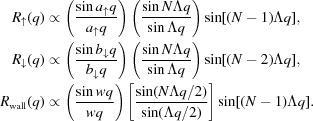
If the diffraction from the three gratings overlaps, the waves are spatially *coherent* with one another, allowing a summation of the amplitudes to deliver the scattered amplitude of the PDI: 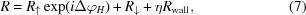
where the phase term is derived from the structure-factor modulation between the 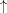 and 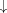 domains, 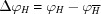. As the amplitude contribution of the wall component is unknown (it is likely to be significantly less than either of the 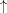 or 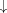 domain contributions as its size 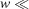 *a* 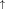 or *b* 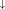), its magnitude is defined by the coefficient η. The spatially coherent diffracted intensity from the PDI 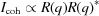. We now consider the possibility that the diffraction from the three gratings does not overlap completely, causing some incoherence in the addition of their amplitudes.

Prior to poling, the sample crystal is single domain, and thus the domain-inverted regions are effectively islands surrounded by a crystal structure of the opposite polarization. It is therefore likely that the 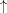 regions are under a strain which is different from that of the 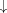 regions, meaning that the average lattice spacing of the inverted domains might be different.

**Figure 11:**
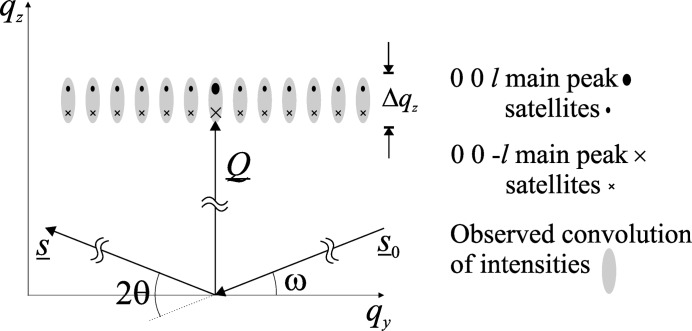
The effect of uneven strains between the adjacent domains in a PDI structure in reciprocal space.

Fig. 11 ▶ shows the effect this would have in reciprocal space on both the main peak and satellites of the two reflections, which become offset by 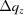. If this offset is less than the Darwin width’s equivalent in *q*-space, or there is actually a distribution of strains (and so lattice spacings), or there is a strong convolution provided by instrumental functions, then the different contributions blur together making them indistinguishable.

We have shown that the LN and KTP samples contained significant strain distributions of 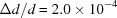 and 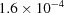, respectively. The adjacent domains may therefore have different (average) lattice spacings, and so diffract the beam in slightly different directions, making the waves spatially *incoherent* with one another. For sufficiently large detector (or analyser) acceptances however (Fig. 11 ▶), the separate contributions will be collected, making a summation of the intensities necessary to deliver the spatially incoherent scattered intensity of the PDI: 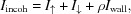
where 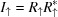 *etc.* and ρ is a coefficient which represents the magnitude of the domain-wall intensity contribution. As each sample is known to contain a distribution of strains, and the analyser acceptance has been shown to be significant, *e.g.* the diagonal streak in the LN 006 map (Fig. 4 ▶), it is likely that each rocking curve contains both coherent and incoherent contributions. Thus, the total reflected intensity from a PDI will therefore be equal to 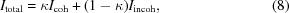
where κ is a coefficient from 0 to 1 which is empirically evaluated.

**Figure 12:**
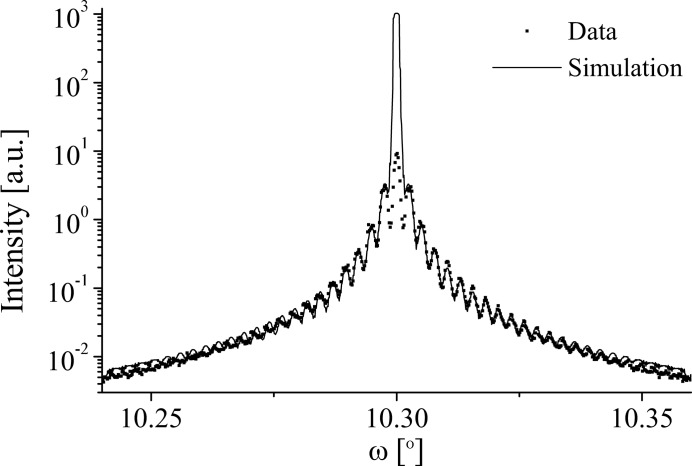
A comparison of the data and grating simulation of the 006 diffraction profile at 15 keV from the 5.01 µm LN sample.

Fig. 12 ▶ shows a comparison of the 5.01 µm LN 006 diffraction profile and simulation (Table 6 ▶). The simulation shows a good fit to the data for all of the satellites, only becoming divergent at the main peak. One possible contribution to this is inadequate dead-time correction of data at high count rates. The simulation was achieved by setting *N* = 2, *D* = 50% (within measured limits), κ = 0 and ρ = 0. 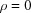 means that no component from the domain walls was included. (In both the coherent and incoherent regimes, the inclusion of a domain-wall component typically boosts the intensity of the even satellites, rather than suppressing it.) The κ value indicates the simulation is entirely incoherent, which is consistent with the significant strain distribution present within the LN grating region, 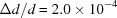, and the relatively long 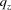 component of the 006 r.l.p., 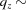 0.85 Å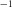. Fig. 9 ▶ shows that as the angle of incidence increases, the region in the strain-sensitive 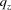 direction sampled by the incident bandwidth, 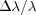, and the region in 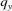 sampled by the detector acceptance, 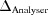, also increase. Thus higher-order reflections are expected to be more convoluted and so less coherent than lower-order reflections. The κ value also means that the profile contains no information concerning the phase shift between the 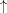 and 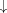 domains, and so it is not possible to obtain atomic level structural information about the domain wall (Table 3 ▶). The *N* value was judged empirically by the widths of the simulated satellites, which become narrower as *N* increases. 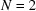 represents a lateral correlation length of 10 µm within the sample, which is well below the coherence length calculated for the 006 reflection, 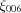 = 122 µm (Table 5 ▶). Thus, it appears that the sample rather than the beam defines the resolution of the diffraction profile.

**Figure 13:**
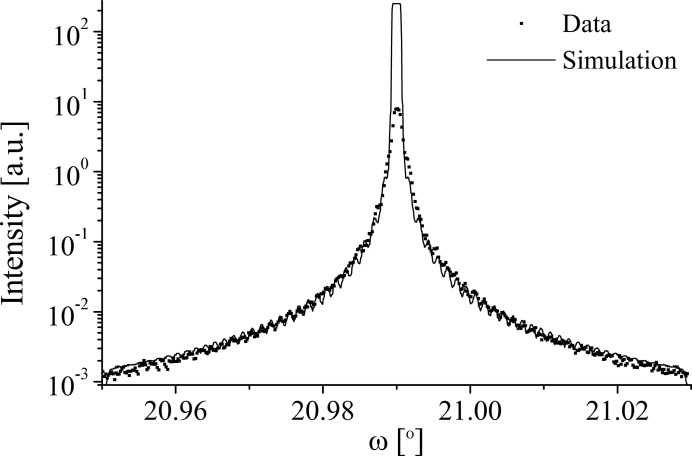
A comparison of the data and grating simulation of the 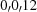 diffraction profile at 15 keV from the 5.01 µm LN sample.

Fig. 13 ▶ shows a comparison of the 5.01 µm LN 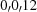 diffraction profile and simulation. The simulation gives a reasonable fit to the data with small satellites of the same magnitude as found in the data being shown. The simulation central peak has a square top which is an artefact introduced by the convolution of a sharp peak with a broad instrumental and sample peak. The simulation was generated by setting *N* = 2, *D* = 50%, κ = 0 and 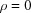. Thus, a component from the domain walls is discounted. As with the LN 006 profile, the simulation is entirely incoherent, ignoring the phase shift of the adjacent domains, and so making it impossible to determine whether Nb or O sits at *z* = 0 between the adjacent domains. Bandwidth and detector acceptance convolutions are more pronounced for higher-order symmetrical Bragg reflections, making this result consistent with the incoherent LN 006 profile. The lateral correlation length within the sample was again identified as 10 µm, well below that of the 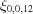 = 61 µm.

**Figure 14:**
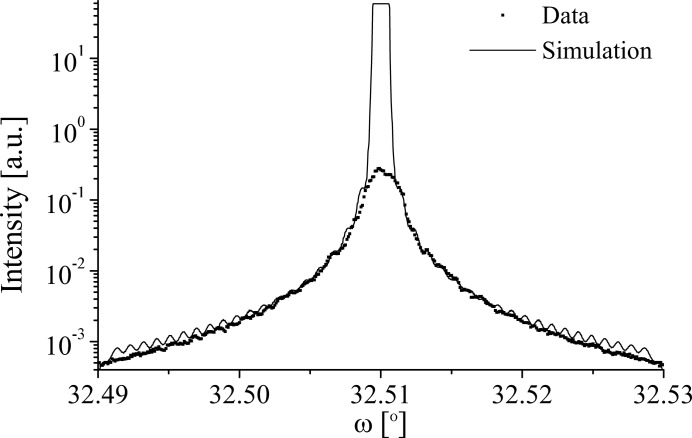
A comparison of the data and grating simulation of the 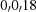 diffraction profile at 15 keV from the 5.01 µm LN sample.

Fig. 14 ▶ shows a comparison of the 5.01 µm LN 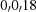 diffraction profile and simulation. The data contain no real features other than a lack of satellites and a characteristic width, both of which are found in the simulation. The square top of the central peak of the simulation comes from the convolution process. The simulation is again incoherent with κ = 0, *N* = 2, *D* = 50% and ρ = 0, meaning no domain-wall component is present. The lateral correlation length is once more given as 10 µm, less than 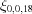 = 41 µm.

**Figure 15:**
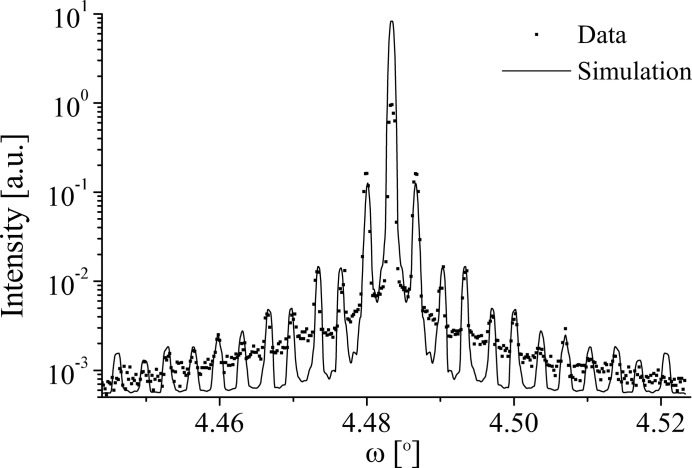
A comparison of the data and grating simulation of the 002 diffraction profile at 15 keV from the 9.02 µm KTP sample.

Fig. 15 ▶ shows a comparison of the 9.02 µm KTP 002 diffraction profile and simulation. The simulation gives a good fit to all the satellite intensities, showing the strong suppression of the even satellites relative to the odd found in the data. The good agreement found between the data and simulation is strong support for the validity of the optical grating model. The simulation was achieved by setting *N* = 7, *D* = 49% (within measured limits), κ = 0.9, 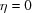 and ρ = 0. Thus, the simulation is almost entirely coherent and contains no domain-wall component. In the coherent regime, the duty cycle was found to control the fall-off of the even satellites, whereas the magnitudes of the odd satellites were given by the phase shift between the 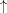 and 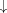 domains, 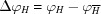. [The incoherent profile is typically monotonic, 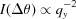.] Though the strains in the KTP and LN samples are very similar in magnitude, 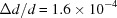 and 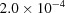, respectively, the lengths of the 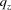 components for the KTP 002 and LN 006 reflections are not: 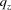 = 0.37 and 0.85 Å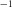, respectively. Both incident wavevectors have the same bandwidth; however the greater the 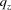 component of each r.l.p., the larger the regions in the 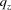 and 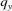 directions sampled around it. Thus the KTP 002 reflection would be expected to be more coherent than the LN 006 reflection, as is found. 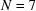 was empirically judged by the satellite widths and gives a lateral correlation length of 63 µm. As with all the LN reflections, this value is below that of the coherence length for the reflection, 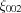 = 278 µm (Table 5 ▶), which suggests again that the sample rather than the beam defines the resolution of the profile. It is possible that the strain distribution 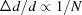; the strain distribution on the grating is an average over the illuminated area, and thus it is probable that as 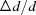 increases, the chances of several adjacent domains containing similar lattice spacings decrease, and *vice versa*. 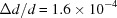 for KTP and 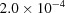 for LN; therefore *N* would be expected to be higher for KTP, as is observed. The most noticeable difference between the simulation and the data is that the simulation consistently falls below the data between satellites. This is attributed to the slit part of the grating model, which is defined by the relative sizes of the 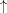 and 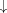 domains, *a* and *b*, respectively. The slit part of the model is not critical to the satellite intensities, but provides a background scattering envelope upon which they sit. Rather than use the slit profile, De Caro *et al.* substitute the reflectivity curve of the crystal 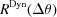. To include 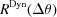 should provide a better fit to the data in the low-intensity regions between the satellites, though it would give no additional information about the domain structure.

**Figure 16:**
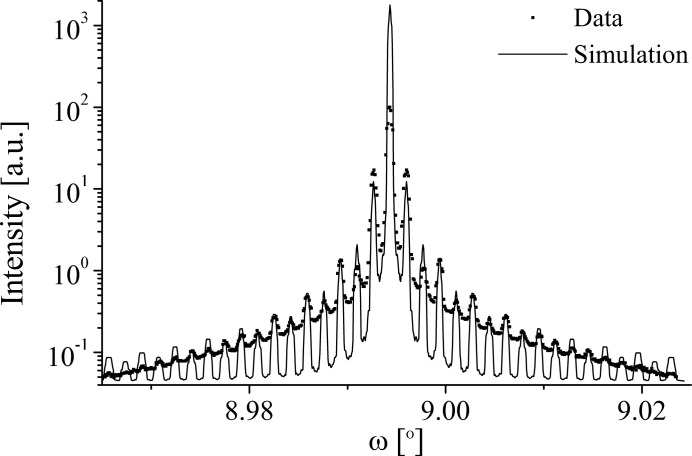
A comparison of the data and grating simulation of the 004 diffraction profile at 15 keV from the 9.02 µm KTP sample.

Fig. 16 ▶ shows a comparison of the 9.02 µm KTP 004 diffraction profile and simulation. The simulation shows a reasonable fit to the data for most of the satellites, showing the strong suppression of the even satellites relative to the odd; however the second and fourth satellite pairs are too high to give a good fit. The simulation was achieved by setting *N* = 7, *D* = 49% (within measured limits), κ = 0.2, η = 0 and ρ = 0. Thus, the simulation is principally incoherent and contains no domain-wall component. For the reasons outlined earlier, the 004 profile would be expected to be more incoherent than the 002 profile. As found before using the coherent model, the odd satellites were controlled by the phase shift between adjacent domains. The simulated odd satellites were significantly lower than the data using 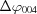, and a good fit could only be achieved by using a phase shift of 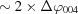 in the coherent model. Typically, the incoherent model provides monotonic satellites such as the LN 006 profile; however here it provides the suppression of even satellites found in the 004 data. The inclusion of coherent and incoherent contributions supports the validity of the optical grating model. The lateral correlation length within the sample was identified as 63 µm in agreement with the 002 reflection.

**Figure 17:**
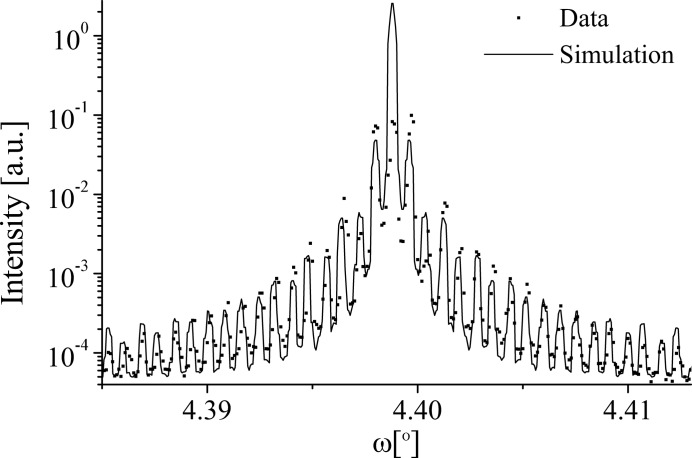
A comparison of the data and grating simulation of the 002 diffraction profile at 15 keV from the 38.85 µm KTA sample.

Fig. 17 ▶ shows a comparison of the 38.85 µm KTA 002 diffraction profile and simulation. The simulation shows a reasonable fit to the data for most of the satellites; however the strong suppression of the second satellite pair is not modelled well by the simulation, and the first and third satellite simulations are below their expected positions. It should be noted, however, that the resolution of the data is low and the simulation does not include any dynamical diffraction effects. The simulation was achieved by setting *N* = 5, *D* = 50% (within measured limits), κ = 0.5, η = 0 and ρ = 0. The simulation consists of equal coherent and incoherent contributions, and no domain-wall component. The problems with the simulation are similar to the problems with the KTP 004 reflection, suggesting the model is not considering all of the factors that influence the profile. The *N* value gives a lateral correlation length within the sample of 194 µm. Although this value is much higher than either that found in the LN and KTP samples, it should be noted that it is still below the coherence length of the beam, 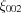 = 284 µm (Table 5 ▶).

## 8. Discussion and conclusions   

The simulations of the HRXD results have been useful in providing an indication of the crystalline state of the LN, KTP and KTA PDI samples. The surface grating model proposed by De Caro *et al.* (1994 ▶) showed that a surface corrugation on the PDI, of the magntitudes found in the literature, would produce satellites in the correct positions; however, their intensities would be far below those observed in the data. Thus, the corrugation depth was discounted as having a significant effect on the satellite intensities. The semi-kinematical model, which described the PDI in terms of a Fourier synthesis and included a domain-wall contribution, was found to be unsuccessful. The reasons for this are likely to include the assumption that both the beam and sample are infinite in extent.

Simulations from the optical grating (OG) model showed reasonable, and in some cases good, agreement with data for all the samples. We emphasize the following points:

(i) As suggested by the BF imaging results, the structure-factor modulation between adjacent domains contributes to the diffraction profiles.

(ii) This picture does not, by itself, adequately model the diffraction data.

The reason for the failure of this simple picture is the need to model the spatial coherence of diffracted waves from adjacent domains. The LN and KTP samples were both found to contain a distribution of strains, 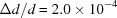 and 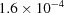, respectively, which in both cases causes the samples to diffract over a range of angles. We propose that 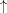 and 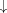 domains have slightly different lattice parameters due to the fact that only the inverted domains have been subjected to a strong electric field (known to cause strain near the surface) and so occupy slightly different regions of reciprocal space. The fact that scattering from the 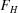 grating can be distinguished from the 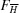 grating introduces another important point:

(iii) Precise determination of the effects of the strain distribution on the scattering of the PDI requires detailed knowledge of the detector acceptance.

Point (iii) suggests that higher-order reflections would be expected to provide more spatially incoherent profiles than lower orders, as borne out by the KTP 002 and 004 and LN 006 and 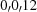 simulations.

It appears probable that a larger strain distribution will have the effect of reducing the lateral correlation length within the sample, *N*, and *vice versa*. *N* was judged empirically from satellite widths and found to be 7 for KTP (63 µm) and 2 for LN (10 µm), supporting the theory that strain distribution within the PDI defines its lateral correlation length, 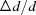 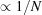. (The width of the KTA reflections in 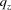 was not measured, so the strain distribution contained in the sample was unknown.) The simulation of the KTA 002 profile showed the lateral correlation length within the sample was 194 µm, and that the profile contained a component of coherent scatter between adjacent domains which produced the even satellite suppression. This suggests the adjacent domains have relatively similar lattice spacings.

The OG model contained a domain-wall contribution, though its inclusion was always found to produce a worse fit. Parameters such as the domain-wall thickness and its scattering properties could therefore not be assessed using this model. The spatial incoherence of the LN profiles also meant it was not possible to define whether the inverted structures in LN were twinned through the Nb or O atoms. The simulations of the KTP 004 and KTA 002 profiles were the least successful and suggest that the OG model is omitting factors which influence the diffraction profile.

As the LN CTR decayed according to 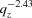, the surface must be relatively imperfect as expected with surface corrugations. This is consistent with the simulations, which show that the LN sample contains absorbing mosaic blocks with a distribution of orientations. BF imaging suggested that the origin of the LN satellites would be the phase shift between adjacent domains. By measuring satellites as a function of energy over an absorption edge, the theory could be assessed. The spatial incoherence of the scattering from the LN sample, however, meant the phase-shift information was lost, making it impossible to verify whether the satellites indeed contained any structural phase information regarding the domain wall in the LN sample. By repeating the measurements over the absorption edge using a miscut analyser, so as to reduce the detector acceptance, it may be possible to show that the satellite intensities are indeed given by the phase shift, and so identify the origin of the twinning in LN as being through Nb, or O.

## Figures and Tables

**Table 1:** Summary of [100] domain-wall properties in KTP

Property	Value	Technique
Latent width	2080nm	AFM (Wittborn *et al.*, 2002 ▶)
Strained width	Unknown	
Corrugation		
*p*-poled	230nm	AFM (Wittborn *et al.*, 2002 ▶)
Single-domain	80nm	AFM (Rommeveaux, 2002 ▶)

**Table 2:** Summary of domain-wall properties in LiNbO
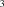

Property	Value	Technique
Latent width	0.20.6m (LT)	NSOM (Yang *et al.*, 1999 ▶)
	515m (LT)	XT (Kim *et al.*, 2000 ▶)
	0.15m	AFM (Wittborn *et al.*, 2002 ▶)
Strained width	3m (LT)	NSOM (Yang *et al.*, 1999 ▶)
Corrugation	2nm	ESFM (Bluhm *et al.*, 1997 ▶)
	0.5nm (LT)	NSOM (Yang Mohideen, 1998 ▶)
	6nm	AFM (Wittborn *et al.*, 2002 ▶)
	10nm	OPSI (Rommeveaux, 2002 ▶)
Shear strain 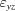	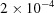 (LT)	NSOM (Yang Mohideen, 1998 ▶)
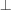 to wall		

**Table 3:** Phase shifts between inverted domains for all reflections investigated from the samples at 15keV

	Friedel pair			Phase difference	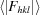
Sample	*h*	*k*	*l*	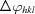 at 15keV ()	(electrons)
LN	0	0	6	63.5	116.2
(Nb at 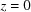 )	0	0	12	34.6	133.0
	0	0	18	20.6	112.8
LN	0	0	6	106.7	116.2
(O at 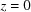 )	0	0	12	51.7	133.0
	0	0	18	30.0	112.8
KTP	0	0	2	106.4	8.0
	0	0	4	53.7	190.4
	0	0	6	5.7	15.2
KTA	0	0	2	131.6	13.2
	0	0	4	43.1	299.0

**Table 4:** Diffraction profile peak widths with diffraction plane normal to the domain walls of all the reflections investigated

				Peak/satellite FWHM (arcsec)			Darwin
	Reflection			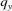 scans		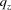 scans	width
Sample	*h*	*k*	*l*	On PDI	Off PDI	On PDI	(arcsec)
LN	0	0	6	4.64	3.07	8.13	2.78
	0	0	12	3.63	2.56		1.69
	0	0	18	2.89	1.55		1.07
KTP	0	0	2	3.03		2.58	0.17
	0	0	4	2.48		5.94	1.90
	0	0	6	1.77			0.11
KTA	0	0	2	0.99			0.25
	0	0	4	0.89			1.92

**Table 5:** Beam coherence for the different reflections investigated in HRXD studies at 15keV

	Reflection			Resolvable	Bragg angle	Eff. 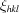
Sample	*h*	*k*	*l*	satellites	()	(m)
LN	0	0	6	Yes	10.3	122
5.01m	0	0	12	No	21.0	61
	0	0	18	No	32.5	41
KTP	0	0	2	Yes	4.5	278
9.02m	0	0	4	Yes	9.0	139
	0	0	6	No	13.6	93
KTA	0	0	2	Yes	4.4	284
38.85m	0	0	4	Yes	8.8	143

**Table 6:** Parameters used in the optical grating model to produce the simulations presented

	Reflection			Degree of coherency	Domain-wall contribution		Periods diffracting
Sample	*h*	*k*	*l*				*N*
LN	0	0	6	0	0		2
5.01m	0	0	12	0	0		2
	0	0	18	0	0		2
							
KTP	0	0	2	0.9	0	0	7
9.02m	0	0	4	0.2	0	0	7
							
KTA	0	0	2	0.5	0	0	5
38.85m							
